# Supplementary material for: Compact graphical representation of phylogenetic data and metadata with GraPhlAn
Source: PeerJ. 2015 Jun 18;3:e1029. doi: 10.7717/peerj.1029 (PMC4476132; doi:10.7717/peerj.1029)
Supplement: Figure S1 — The cladogram shows the aerobiosis analysis of the HMP data in three O2-dependent classes: aerobic (red), anaerobic (blue), and microaerobic (green). The node size reflects the abundance level of each clades, colors are assigned accordingly to one of the three classes, while the lightness intensity of colors respect the LDA score assigned by LEfSe to biomarkers. Data used for this image is available as indicated under “Datasets used” paragraph in “Materials and Methods” section. [file peerj-03-1029-s001.pdf]

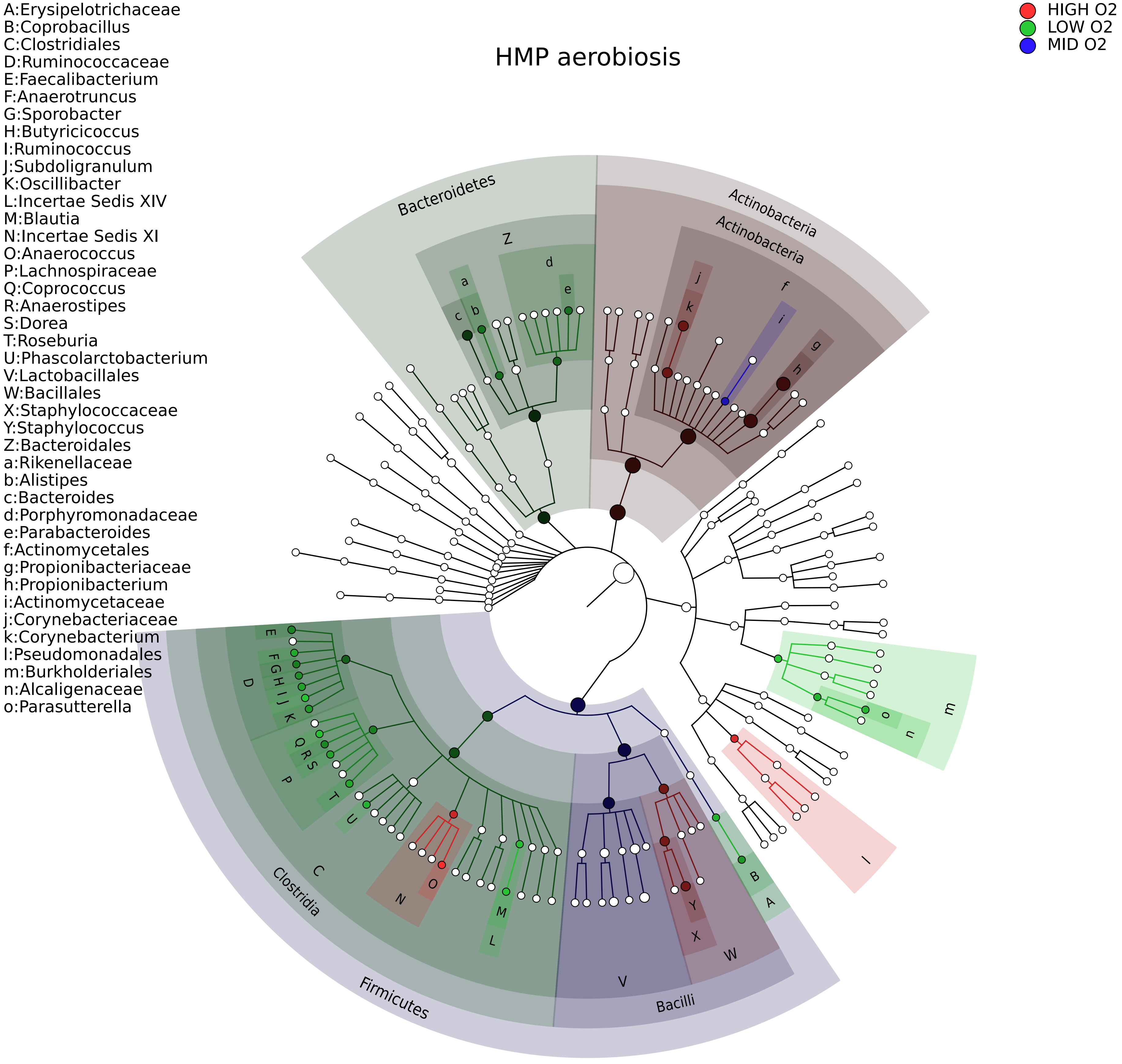
HMP aerobiosis
HIGH O2
LOW O2
MID O2
A:Erysipelotrichaceae
B:Coprobacillus
C:Clostridiales
D:Ruminococcaceae
E:Faecalibacterium
F:Anaerotruncus
G:Sporobacter
H:Butyricicoccus
I:Ruminococcus
J:Subdoligranulum
K:Oscillibacter
L:Incertae Sedis XIV
M:Blautia
N:Incertae Sedis XI
O:Anaerococcus
P:Lachnospiraceae
Q:Coprococcus
R:Anaerostipes
S:Dorea
T:Roseburia
U:Phascolarctobacterium
V:Lactobacillales
W:Bacillales
X:Staphylococcaceae
Y:Staphylococcus
Z:Bacteroidales
a:Rikenellaceae
b:Alistipes
c:Bacteroides
d:Porphyromonadaceae
e:Parabacteroides
f:Actinomycetales
g:Propionibacteriaceae
h:Propionibacterium
i:Actinomycetaceae
j:Corynebacteriaceae
k:Corynebacterium
l:Pseudomonadales
m:Burkholderiales
n:Alcaligenaceae
o:Parasutterella
Bacteroidetes
Actinobacteria
Actinobacteria
Firmicutes
Clostridia
Bacilli
