# Supplementary material for: Compact graphical representation of phylogenetic data and metadata with GraPhlAn
Source: PeerJ. 2015 Jun 18;3:e1029. doi: 10.7717/peerj.1029 (PMC4476132; doi:10.7717/peerj.1029)
Supplement: Figure S2 — This image shows the taxonomic enrichment of the first saliva microbiome sequenced using IonTorrent PGM technology. We exploit export2graphlan capability of handle BIOM files to generate the annotation and tree files for GraPhlAn. Data used for this image is available as indicated under “Datasets used” paragraph in “Materials and Methods” section. [file peerj-03-1029-s002.pdf]

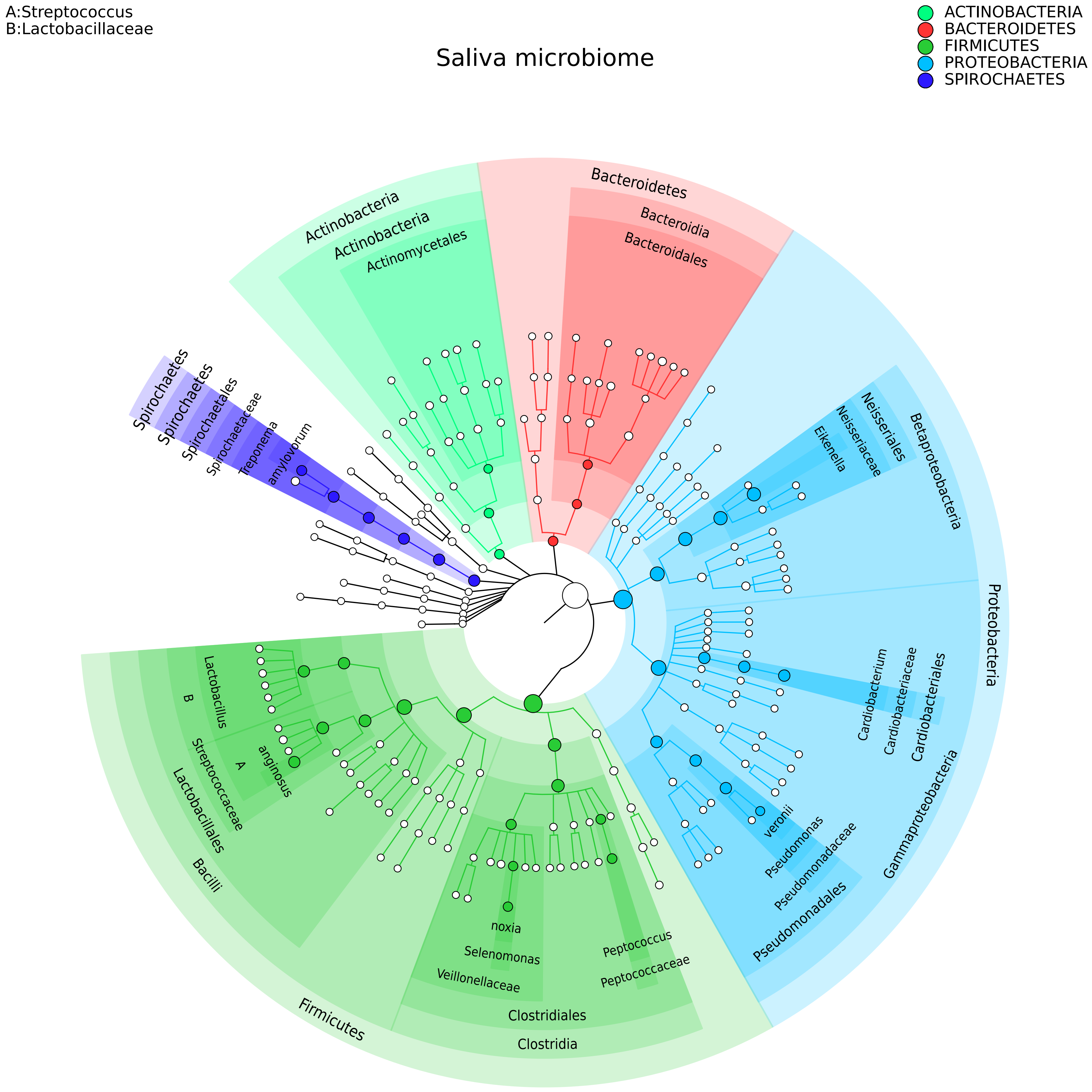
A:Streptococcus
B:Lactobacillaceae
Saliva microbiome
ACTINOBACTERIA
BACTEROIDETES
FIRMICUTES
PROTEOBACTERIA
SPIROCHAETES
Actinobacteria
Actinobacteria
Actinomycetales
Bacteroidetes
Bacteroidia
Bacteroidales
Spirochaetes
Spirochaetes
Spirochaetales
Spirochaetaceae
Treponema
amylovorum
Neisseriales
Neisseriaceae
Eikenella
Betaproteobacteria
Proteobacteria
Cardiobacterium
Cardiobacteriaceae
Cardiobacteriales
Gammaproteobacteria
veronii
Pseudomonas
Pseudomonadaceae
Pseudomonadales
Peptococcus
Peptococcaceae
noxia
Selenomonas
Veillonellaceae
Clostridiales
Clostridia
Firmicutes
Bacilli
Lactobacillales
Streptococcaceae
A
anginosus
B
Lactobacillus
