# Supplementary material for: Compact graphical representation of phylogenetic data and metadata with GraPhlAn
Source: PeerJ. 2015 Jun 18;3:e1029. doi: 10.7717/peerj.1029 (PMC4476132; doi:10.7717/peerj.1029)
Supplement: Figure S3 — We take the data as a BIOM file from the (Gajer et al., 2012) study. We use export2graphlan to generate the needed files for plotting the circular tree with GraPhlAn. Data used for this image is available as indicated under “Datasets used” paragraph in “Materials and Methods” section. [file peerj-03-1029-s003.pdf]

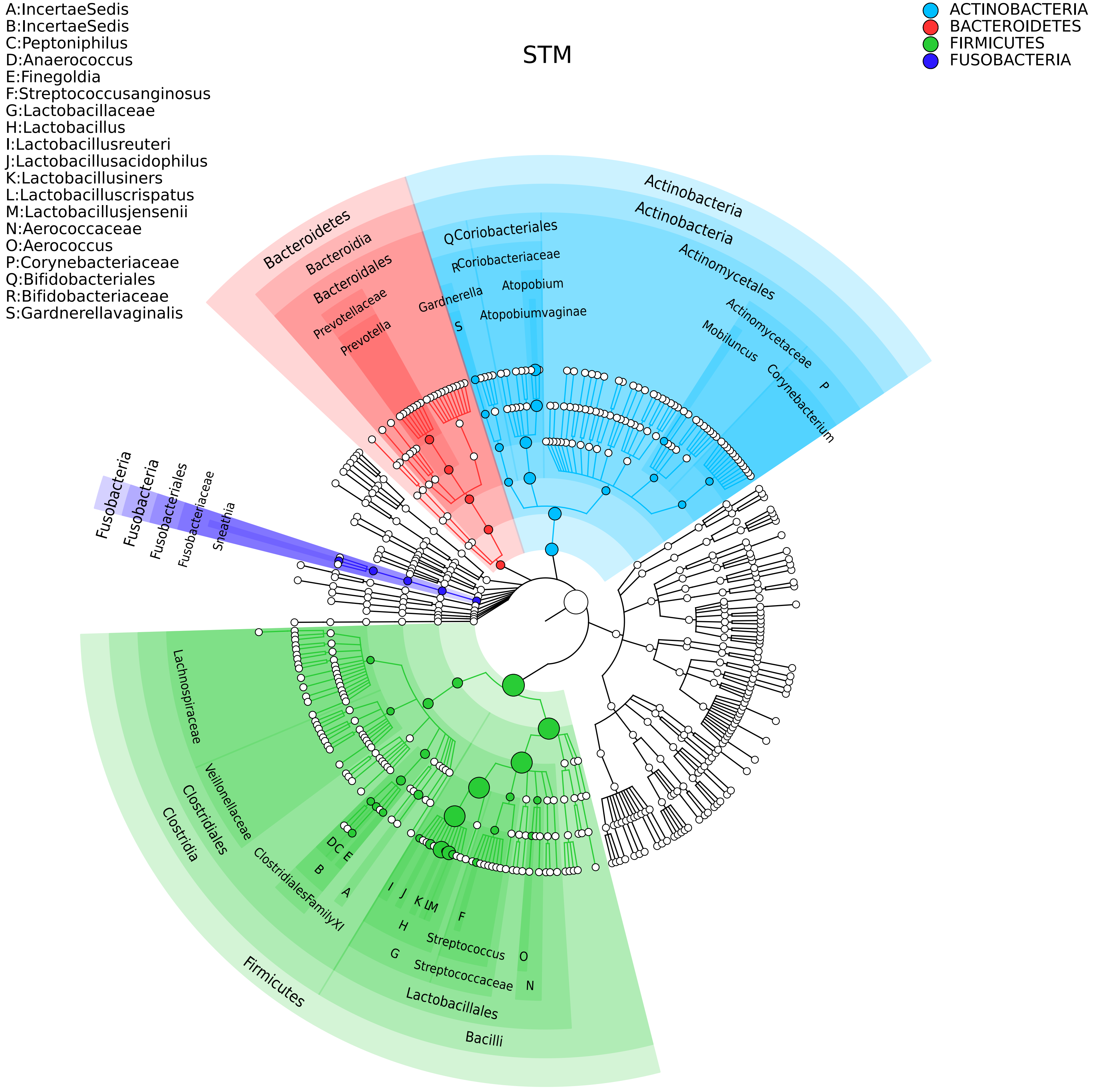
STM
A:IncertaeSedis
B:IncertaeSedis
C:Peptoniphilus
D:Anaerococcus
E:Finegoldia
F:Streptococcusanginosus
G:Lactobacillaceae
H:Lactobacillus
I:Lactobacillusreuteri
J:Lactobacillusacidophilus
K:Lactobacillusiners
L:Lactobacilluscrispatus
M:Lactobacillusjensenii
N:Aerococcaceae
O:Aerococcus
P:Corynebacteriaceae
Q:Bifidobacteriales
R:Bifidobacteriaceae
S:Gardnerellavaginalis
ACTINOBACTERIA
BACTEROIDETES
FIRMICUTES
FUSOBACTERIA
Actinobacteria
Actinobacteria
Actinomycetales
Actinomycetaceae
Mobiluncus
Corynebacterium
P
QCoriobacteriales
RCoriobacteriaceae
Atopobium
Atopobiumvaginae
Gardnerella
S
Bacteroidetes
Bacteroidia
Bacteroidales
Prevotellaceae
Prevotella
Fusobacteria
Fusobacteria
Fusobacteriales
Fusobacteriaceae
Sneathia
Lachnospiraceae
Veillonellaceae
Clostridiales
Clostridia
ClostridialesFamilyXI
DC E
B
A
I J K LM
F
H
Streptococcus
O
G
Streptococcaceae
N
Lactobacillales
Bacilli
Firmicutes
